# Fragile X Syndrome Diagnosis Patterns in a Multistate, Population-Based Cohort

**DOI:** 10.1001/jamanetworkopen.2026.42086

**Published:** 2026-11-06

**Authors:** Manish Ghimire, Michelle Curtin, Stephen Downs, Arezoo Movaghar

**Affiliations:** 1 Department of Pediatrics, Wake Forest University School of Medicine, Winston-Salem, North Carolina; 2 Department of Biomedical Engineering, Wake Forest University School of Medicine, Winston-Salem, North Carolina

## Abstract

This cross-sectional study of a multistate cohort examines rates of fragile X syndrome diagnosis by sex and race.

## Introduction

Fragile X syndrome (FXS) is an inherited, multisystem genetic disorder associated with substantial clinical and socioeconomic burden.^1,2^ Early and accurate diagnosis is critical for access to appropriate care, services, and genetic counselling. However, FXS is not included in standard newborn screening panels, and recommendations for genetic testing are often not fully implemented.^3^ Prior work on a predominantly White Wisconsin population suggested that more than 67% of individuals with FXS remain undiagnosed.^4^ However, diagnostic patterns in the broader FXS population are unknown. The current study aims to provide population-level estimates of FXS diagnosis in a multistate cohort of more than 7.3 million patients.

## Methods

This cross-sectional study was conducted using electronic health records (EHR) from Atrium Health, a large health system providing care to patients in 4 states: Alabama, Georgia, North Carolina, and South Carolina. Patients’ medical data from 1973 to 2026, including diagnostic codes, sex, and self-reported race were obtained. Individuals with FXS were identified using diagnostic codes. *FMR1* premutation carriers, identified through manual review of records, were excluded. To improve diagnostic specificity and to reduce the possibility of rule-out tests, only individuals with at least 2 FXS encounters were included. The expected number of cases was calculated using prevalence estimates reported by Hunter et al^5^ as 1.4 per 10 000 male and 0.9 per 10 000 female individuals (eAppendix 1 in Supplement 1).^6^ Although these estimates are based on studies predominantly conducted in White populations, research on *FMR1* premutation has shown no significant differences in prevalence between Black and White patients.^7^ Additionally, phenotyping studies showed similar health profiles between these 2 populations.^8^ The diagnosis rate, defined as the ratio of clinically diagnosed cases to expected cases based on population prevalence estimates, was calculated for the overall population, stratified by sex and race. Observed and expected rates were compared using 1-sided population proportion tests, and between-group comparisons were performed using 2-sided tests, with *P* < .05 as a threshold for statistical significance (eAppendix 2 in Supplement 1).^4^ This study followed the Strengthening the Reporting of Observational Studies in Epidemiology (STROBE) reporting guideline and was approved by the institutional review board of Wake Forest University School of Medicine. Only deidentified EHR data were used and therefore informed consent was waived. Analyses were conducted using Python version 3.12 (Python Software Foundation) and concluded in August 2026.

## Results

Data from 7 381 668 patients who self-reported as Black or African American (1 774 770 patients; 947 033 female [53.3%]) or White (5 606 898 patients; 2 932 020 female [52.3%]) were available (Table 1). The overall expected number of cases was estimated to be 839 patients (201 Black and 638 White individuals). However, only 231 patients (36 Black and 195 White individuals) received an FXS diagnosis, indicating a substantial gap between the number of diagnosed cases and the expected number of patients. The rate at Atrium Health was lower but not significantly different from our previous study in Wisconsin (27.5% [95% CI, 24.1%-31.2%] vs 32.6%; *P* = .06).^4^ The overall diagnosis rate in Black population was 17.9% (95% CI, 12.4%-24.4%), which is significantly lower than the White population (30.6% [95% CI, 26.3%-35.1%]; *P* < .001) (Table 2). The estimated underdiagnosis rate in women was higher than in men (diagnosis rate: all women, 18.3% [95% CI, 14.0%-23.2%] vs all men, 34.1% [95% CI, 29.0%-39.6%]; *P* < .001; Black women, 3.5% [95% CI, <0.1%-9.4%] vs Black men, 28.5% [95% CI, 19.0%-39.7%]; *P* < .001; White women, 23.1% [95% CI, 17.4%-29.6%] vs White men, 35.8% [95% CI, 30.0%-42.3%]; *P* < .001). Even though the diagnosis rate was lower in Black men, no significant difference was observed compared with White men (28.5% [95% CI, 19.0%-39.7%] vs 35.8% [95% CI, 30.0%-42.3%]; *P* = .14). However, Black women had a significantly higher underdiagnosis rate compared with White women (3.5% [95% CI, <0.1%-9.4%] vs 23.1% [95% CI, 17.4%-29.6%]; *P* < .001).

**Table 1.** Diagnosis Rate of Fragile X Syndrome in Atrium Health System^a^

Group	Total patients	Cases		Diagnosis rate, % (95% CI)	*P* value
		Diagnosed	Expected		
All	7 381 668	231	839	27.5 (24.1-31.2)	<.001
Male	3 502 615	167	490	34.1 (29.0-39.6)	<.001
Female	3 879 053	64	349	18.3 (14.0-23.2)	<.001
Black	1 774 770	36	201	17.9 (12.4-24.4)	<.001
Male	827 737	33	116	28.5 (19.0-39.7)	<.001
Female	947 033	3	85	3.5 (<0.1-9.4)	<.001
White	5 606 898	195	638	30.6 (26.3-35.1)	<.001
Male	2 674 878	134	374	35.8 (30.0-42.3)	<.001
Female	2 932 020	61	264	23.1 (17.4-29.6)	<.001

^a^ The diagnosis rate is defined as the ratio of number of diagnosed cases to number of expected cases. 95% CIs were calculated using the exact Poisson method.

**Table 2.** Comparison of Fragile X Syndrome Diagnosis Rates Across Patient Subgroups

Groups	*Z*-statistic	*P* value
Black all vs White all	3.50	<.001
Black male vs White male	1.47	.14
Black female vs White female	4.06	<.001
Black female vs Black male	4.55	<.001
White female vs White male	3.44	<.001
All female vs all male	5.03	<.001

## Discussion

FXS is consistently underdiagnosed across geographical regions, clinical settings, and patient populations. Although men are significantly underdiagnosed in the general population, Black and White men had similar diagnosis rates. Women had a higher underdiagnosis rate compared with men, with a significant gap in diagnosis between White and Black women. These disparities could be partly due to the fact that FXS in men is often more severe, with recognizable clinical manifestations. In contrast, women frequently exhibit milder or more variable phenotypes, which may reduce clinical recognition and contribute to the higher underdiagnosis rates. The particularly high underdiagnosis rate observed among Black women may also reflect the persistent disparities in access to developmental, genetic, and specialty care.^9,10^ Overall, these findings highlight an urgent need for improved screening and diagnostic approaches, particularly for individuals who do not present with the severe or classic phenotypes. Additionally, expanding access to genetic testing and specialty care among underrepresented populations should be a public health priority. The findings should be interpreted in the context of important limitations. Data span over 5 decades, during which changes in clinician knowledge, genetic testing, and clinical practices may have influenced case ascertainment and documentation. The detection of cases relies on diagnostic codes and further clinical verification and validation across health systems and clinical practices are needed. Finally, the true prevalence of FXS is unknown and the reported rates are calculated based on current prevalence estimates. These factors warrant further investigation and should be considered when interpreting the results of this study.

## Notes

Supplement 1. eAppendix 1. Wisconsin population eAppendix 2. Statistical methods eReferences

Supplement 2. Data sharing statement
