# Supplementary material for: Fragile X Syndrome Diagnosis Patterns in a Multistate, Population-Based Cohort
Source: JAMA Netw Open. 2026 Nov 6;9(11):e2642086. doi: 10.1001/jamanetworkopen.2026.42086 (PMC13744017; doi:10.1001/jamanetworkopen.2026.42086)
Supplement: Supplementary file 1 [file jamanetwopen-e2642086-s001.pdf]

Supplemental Online Content

Ghimire M, Curtin M, Downs S, Movaghar A. Fragile X syndrome diagnosis patterns in a multistate population-based cohort. *JAMA Netw Open.* 2026;9(11):e2642086. doi:10.1001/jamanetworkopen.2026.42086

eAppendix 1. Wisconsin population

eAppendix 2. Statistical methods

eReferences

This supplemental material has been provided by the authors to give readers additional information about their work.

© 2026 Ghimire M et al. *JAMA Network Open*.

eAppendix 1. Wisconsin Population

To compare diagnosis rates with the prior Wisconsin study, the two cohorts reported by Movaghar et al. (2021) were combined[1]. The prevalence estimates used to calculate the expected cases were 1.4 per 10,000 males and 0.9 per 10,000 females as reported by Hunter et al. (2014)[2]. UW Health (total patients: 2,084,289; clinically diagnosed FXS cases: 87; expected cases: 239) and Marshfield Clinic (total patients: 1,723,223; clinically diagnosed FXS cases: 55; expected cases: 196) were pooled by summing diagnosed cases (n=142) and expected cases (n=435). The diagnosis rate was calculated as the number of clinically diagnosed cases divided by the expected number of cases. The combined Wisconsin diagnosis rate was estimated to be 32.6% (40.6% among males, 21.2% among females). Between-group comparisons between Atrium Health and the combined Wisconsin cohort were performed.

© 2026 Ghimire M et al. *JAMA Network Open*.

eAppendix 2. Statistical Methods

Exact Poisson 95% confidence intervals (CIs) were calculated for all diagnosis rates using Python's scipy.stats.poisson module, following the method described by Ulm (1990)[3].

For an observed count x and expected number of cases E, the CI bounds were calculated as:

$$\text{Lower bound} = \frac{F_{\text{Poisson}}^{-1}(\frac{\alpha}{2}, x)}{E} \times 100\%$$

$$\text{Upper bound} = \frac{F_{\text{Poisson}}^{-1}(1 - \frac{\alpha}{2}, x + 1)}{E} \times 100\%$$

where F_{Poisson}^{-1} represents the inverse Poisson distribution and $\alpha = 0.05$.

© 2026 Ghimire M et al. *JAMA Network Open*.

eReferences

1. Movaghar A, Page D, Brilliant M, Mailick M. Prevalence of Underdiagnosed Fragile X Syndrome in 2 Health Systems. *JAMA Netw Open*. 2021;4(12):e2141516. doi:10.1001/jamanetworkopen.2021.41516

2. Hunter J, Rivero-Arias O, Angelov A, Kim E, Fotheringham I, Leal J. Epidemiology of fragile X syndrome: A systematic review and meta-analysis. *American J of Med Genetics Pt A*. 2014;164(7):1648-1658. doi:10.1002/ajmg.a.36511

3. Ulm K. A simple method to calculate the confidence interval of a standardized mortality ratio (SMR). *Am J Epidemiol*. 1990;131(2):373-375. doi:10.1093/oxfordjournals.aje.a115507

© 2026 Ghimire M et al. *JAMA Network Open*.
