# Supplementary material for: Fragile X Syndrome Diagnosis Patterns in a Multistate, Population-Based Cohort
Source: JAMA Netw Open. 2026 Nov 6;9(11):e2642086. doi: 10.1001/jamanetworkopen.2026.42086 (PMC13744017; doi:10.1001/jamanetworkopen.2026.42086)
Supplement: Supplementary file 2 [file jamanetwopen-e2642086-s002.pdf]

Data Sharing Statement

Data
Data available: Yes
Data types: Other (please specify)
Additional Information: Anonymized data will be made available via direct request to the authors and with appropriate data use agreements with Wake Forest University School of Medicine and Atrium Health System.
How to access data: arezoo.movaghar@wfusm.edu
When available: With publication

Supporting Documents
Document types: None

Additional Information
Who can access the data: Researchers whose proposed use of the data has been approved
Types of analyses: Research
Mechanisms of data availability: with a signed data access agreement
